# Perspectives of Clients and Health Care Professionals on the Opportunities for Digital Health Interventions in Cerebrovascular Disease Care: Qualitative Descriptive Study

**DOI:** 10.2196/52715

**Published:** 2024-12-02

**Authors:** Henna Härkönen, Kirsi Myllykangas, Mikko Kärppä, Kirsi Maaria Rasmus, Julius Francis Gomes, Milla Immonen, Piia Hyvämäki, Miia Jansson

**Affiliations:** 1 Research Unit of Health Sciences and Technology (HST) University of Oulu Oulu Finland; 2 Neurocenter Department of Neurology Oulu University Hospital Oulu Finland; 3 Research Unit of Clinical Medicine University of Oulu Oulu Finland; 4 Faculty of Medicine University of Oulu Oulu Finland; 5 Oulu Business School University of Oulu Oulu Finland; 6 VTT Technical Research Centre of Finland Ltd Oulu Finland; 7 College of Science, Technology, Engineering and Mathematics (STEM) RMIT University Melbourne Australia

**Keywords:** cerebrovascular disease, stroke, digitalization, interventions, health care professional, client, patient, mHealth, mobile health, application, digital health, smartphones, health system, qualitative, descriptive study, brain, blood vessel disease, cerebrovascular disorder, Finland, interviews, efficiency, information, quality, accountability, neurology, neuroscience, brain injury, mobile phone

## Abstract

**Background:**

Cerebrovascular diseases (CVDs) are a major and potentially increasing burden to public health. Digital health interventions (DHIs) could support access to and provision of high-quality health care (eg, outcomes, safety, and satisfaction), but the design and development of digital solutions and technologies lack the assessment of user needs. Research is needed to identify opportunities to address health system challenges and improve CVD care with primary users of services as the key informants of everyday requirements.

**Objective:**

This study aims to identify opportunities for DHIs from clients’ and health care professionals’ perspectives to address health system challenges and improve CVD care.

**Methods:**

This study used a qualitative, descriptive approach. Semistructured, in-person interviews were conducted with 22 clients and 26 health care professionals in a single tertiary-level hospital in Finland between August 2021 and March 2022. The data were analyzed using a deductive and inductive content analysis.

**Results:**

Identified opportunities for DHIs in CVD care were organized according to clients, health care professionals, and data services and classified into 14 main categories and 27 generic categories, with 126 subcategories of requirements. DHIs for clients could support the long-term management of health and life changes brought on by CVD. They could provide access to personal health data and offer health information, support, and communication possibilities for clients and their caregivers. Health care professionals would benefit from access to relevant patient data, along with systems and tools that support competence and decision-making. Intersectoral and professional collaboration could be promoted with digital platforms and care pathways. DHIs for data services could enhance care planning and coordination with novel predictive data and interoperable systems for data exchange.

**Conclusions:**

The combined study of client and health care professional perspectives identified several opportunities and requirements for DHIs that related to the information, availability, quality, acceptability, utilization, efficiency, and accountability challenges of health systems. These findings provide valuable social insights into digital transformation and the emerging design, development, and use of user-centered technologies and applications to address challenges and improve CVD care and health care.

## Introduction

Cerebrovascular diseases (CVDs) are a group of neurological conditions such as strokes (brought on by ischemia or hemorrhage) and transient ischemic attacks (TIAs) [1]. CVDs are one of the major burdens to public health globally as strokes are a leading cause of mortality and long-term disability [2]. The average lifetime costs of stroke are around $66,956-$74,605 [3]. The total costs of CVD are, however, estimated to rise by the year 2047 due to improved stroke survival rates and the aging population [4].

The use of digital health interventions (DHIs) has increased rapidly during the last decade [5] to tackle health system challenges, with the potential to increase the quality, accessibility, and availability of health services [6]. Current health care services face challenges in delivering high-quality primary [7] and secondary prevention [8], rehabilitation [9], and long-term support [10-12] for CVD. The European Stroke Action Plan emphasizes the research and development of digital systems, especially to support patients’ self-management in CVD to reduce the risk of strokes and increase the quality of care [13].

DHIs have shown promise in the primary prevention of CVD with their growing acceptability and use by clients and health care professionals [14]. They can increase the availability of customized health and risk information and increase the self-management capabilities (eg, mobility, positive emotions, and behavior) of patients with CVD [15]. Pre- and in-hospital specialized telestroke services (eg, remote neurological examinations, triage, and case management) are efficient at improving acute CVD care and subsequent health outcomes [16]. In addition, individually tailored delivery of telerehabilitation, with the opportunity to view their own progress, can engage patients with an improved sense of control, motor skills, and activity [17].

Clients usually have positive attitudes and acceptance toward DHIs [5]. Patients with CVD value the provided benefits of the availability of quality information and access to their own health data, supportive supervision of rehabilitation [18], connection to people, and managing everyday life [19]. However, the impact of DHIs is affected by internal (eg, motivation and social challenges) and external (eg, practical and technical barriers) factors [17], as well as cognitive and functional challenges hindering usage [19].

Health care professionals are members of the health workforce who deliver health services [20]. They generally value DHIs as improving communication with clients and other health care professionals, with improvements for care planning, coordination, and workflow management [21]. DHIs that meet health care professional requirements are also vital, as poor usability and functionality add stress and reduce motivation for use [22]. Health care professionals do see the necessity of digitalization for improving CVD care [23], but little is known about the various requirements they have for DHIs to deliver high-quality care, especially in the acute CVD context.

Previous research has identified that DHIs are seldom designed from the primary user perspective to address specific health needs [24], which can lead to potential health disparities and reduce benefits gained [25]. Therefore, more qualitative research is required to identify how technology can address health system challenges from both the client and health care professional perspectives.

The aim of this study was to identify opportunities for DHIs from the client and health care professional perspectives to address health system challenges and improve care in CVD. The research question was as follows: What kind of requirements for DHIs do clients and health care professionals have in CVD care?

## Methods

### Study Design

A qualitative, descriptive, phenomenological approach was used with semistructured interviews and content analysis techniques to describe client and health care professional primary user perspectives of DHIs in CVD care [26]. The study was conducted in one of the major tertiary-level hospitals in Finland providing comprehensive acute CVD care. The hospital is located in the geographically largest hospital district area for specialized medical care in Finland [27] with a high prevalence of CVD [28]. Finland is one of the leading countries in digitalization, with high national eHealth maturity in health care [29] and a national information system service (Kanta Services) with archiving of electronic patient data [30].

### Recruitment

The participants were recruited with convenience sampling. The inclusion criteria for the patients were as follows: adults (aged 18 years and older) with diagnosed ischemic stroke or TIA (I63.0-63.9, G45.0-G45.9, I60, or I61.0-61.9), an in-patient at the acute neurological ward, and able to give an informed consent. Patients were first assessed for eligibility by the ward staff. If eligible, patients were approached by a study nurse (RL) after obtaining preliminary informed consent. Patient refusals (n=62) were related to the patient’s assessment of not being able to answer the questions or not feeling well enough, length of interview, or lack of time due to an upcoming hospital discharge.

The inclusion criteria for the health care professionals were adults (aged 18 years or older) directly involved in CVD care at the acute neurological ward or at a CVD diagnostic and rehabilitation ward. The criteria were broad to ensure the representation of the various professionals involved in CVD care. The health care professionals were contacted face-to-face or by email for recruiting by the study nurse. The number of refusals was not documented, but high workload was the most common reason for refusals.

### Data Collection

Data were collected between August 2021 and March 2022 to recruit enough patients and health care professionals to comprehensively answer the research question. Participants were recruited until data saturation [26]. Semistructured individual interviews were conducted once in Finnish by an experienced study nurse (RL, registered nurse) in a private room at the hospital. The study nurse had no prior relationship with the participants. Separate topic guides were used for participants based on previous studies [31-33]. The interviews were recorded, with no additional field notes made. The duration of the clients’ interviews varied from 19 to 72 minutes and the duration of the health care professional interviews varied from 11 to 89 minutes.

### Data Analysis

The interview transcripts were coded and categorized using NVivo qualitative data analysis software (version 1.6.1; QRR International Pty Ltd). The data were first sorted into open codes with a unit of analysis being a thought pattern [34]. In accordance with the World Health Organization (WHO) categorization [20], opportunities for DHIs were deductively organized according to overarching groupings based on the targeted primary user (clients, health care providers, health system managers, and data services) and classified according to main categories and generic categories [35]. The subcategories (ie, requirements for DHIs) were then defined inductively [34]. A categorization example is presented in Table 1. The analysis with all aforementioned stages was conducted by the corresponding author (HH) and verified by authors (KM and MJ) with methodological and research expertise.

**Table 1:** Example of the content analysis categorization and related quotations from the interview data.

Main category and generic category			Subcategory		Examples of quotations
**Targeted client communication**					
	Health information	Accessibility		H20:* …video content and all that. Reading all that text and picking things up and adopting them can be so limited, that it would be easier if someone would speak it. And it would be easily available at that moment when you are alert…* H25:* …An app or a computer system, where you can straightforwardly move things forward. That will guide it step by step. But I think they should be in a figurative form with only a few things at a time. Because CVD patients often have that difficulty in comprehension…simple with also that picture as a clue, not necessarily only verbal. I think, it is right for that building of everyday life, especially at the hospital discharge phase it would be important.* P19:* They could come to your phone, for example a counselling video to your e-mail or something. That would be easy to watch it from there then. And would be easier to read than some centimeter thick booklet on the same matter that could be seen on video in 5 to 10 minutes.*	
	Alerts and reminders	Daily tasks		H16:* Of course, I wish that there could also be those types of daily tasks and all, like have you thought about this and done that…eat this and a tip of the day and stretch. Do not drink alcohol today.* P9:* Of course, the application could be like, it is 16 and a half degrees warm, sun is shining, go for a walk.* P1: *You could always add some kind of exercise application, a pedometer and so forth…that nags if you have not exercised enough.*	

### Ethical Considerations

The study was conducted according to the national guidelines for good ethical conduct [36]. Approvals were obtained from the Ethics Committee of Northern Ostrobothnia Hospital District (Decision No: 46/2021) and from the Northern Ostrobothnia Hospital District Board (Decision No: 30/2021). Written consent was obtained prior to the interviews. Anonymity was ensured by using ID numbers. All research data were stored in digital password-protected files. Any data used in this paper have been lawfully acquired in accordance with the Nagoya Protocol on Access to Genetic Resources and the Fair and Equitable Sharing of Benefits Arising from Their Utilization to the Convention on Biological Diversity.

### Rigor and Reflexivity

The participants were purposefully selected and recruited until data saturation to improve credibility [26] with an appropriate sample size of informants concerning the phenomenon. The analysis was verified by 2 researchers with results discussed in the research group, thus establishing dependability. In addition, the first author had access to only ID-numbered transcripts with no prior connection to the participants, which reduced bias. Confirmability and dependability were ensured by comparing transcripts with the findings to ensure the representation of the participants’ views and verify the process. Several quotations from various participants were provided to represent authenticity. The Consolidated Criteria for Reporting Qualitative Research (COREQ) [37] has been used to provide reliable reporting (Multimedia Appendix 1). The demographic characteristics of participants are provided to improve transferability (Table 2).

**Table 2:** Demographic characteristics of the study participants.

Characteristic		Clients (n=22), n (%)	Health care professionals (n=26), n (%)
**Sex**			
	Female	7 (32)	21 (81)
	Male	15 (68)	5 (19)
**Age group (years)**			
	20-39	2 (9)	14 (54)
	40-59	8 (36)	12 (46)
	60-79	7 (32)	0 (0)
	80 and older	5 (23)	0 (0)
**Highest academic degree**			
	Primary education	8 (36)	0 (0)
	Vocational training	9 (41)	3 (12)
	Undergraduate degree	5 (23)	10 (38)
	Graduate degree	0 (0)	13 (50)
**Working status**			
	Student	1 (5)	N/A^a^
	Employee or entrepreneur	9 (41)	N/A
	Retired	12 (55)	N/A
**Profession**			
	Practical care nurses	N/A	4 (15)
	Registered nurses	N/A	9 (35)
	Physiotherapist or speech therapists	N/A	3 (12)
	Physician or psychologists	N/A	10 (38)
**Prior cerebrovascular symptoms**			
	Yes	6 (27)	N/A
	No	16 (73)	N/A

^a^N/A: not applicable or available.

## Results

### Characteristics of Participants

A total of 46 participants were interviewed (Table 2). Most (16/22, 73%) of the clients have recently experienced their first CVD. The health care professionals were physicians or psychologists who specialized in neurology or neuropsychology, registered nurses, practical care nurses, and physiotherapists or speech therapists.

Identified opportunities for DHIs in CVD were organized according to the clients, health care professionals, and data services, and they were classified into 14 main categories, 27 generic categories, and 126 subcategories. The identified opportunities for DHIs were related to information, availability, quality, acceptability, utilization, efficiency, and accountability of health systems.

### Clients

The opportunities of DHIs for clients were classified under 5 main categories: targeted communication, untargeted communication, peer communication, personal health tracking, and on-demand information services (Table 3).

**Table 3:** Identified opportunities and requirements of digital health interventions for clients in cerebrovascular disease care.

Main category and generic category			Subcategory
**Targeted client communication**			
	Health information	Evidence-based information Availability Accessibility Adaptability Tailorability Visualization of care pathway Multimedia content Personalized tasks and activities Convenient timing Involving caregivers Multimodality	
	Alerts and reminders	Contacting client groups Color coding of reference values Motivation support Games Customized message intervals Personalization Daily tasks Informing caregivers	
	Diagnostic results	Mobile access Long-term follow-up	
**Untargeted client communication**			
	Untargeted health information	Multimodality	
**Client-to-client communication**			
	Peer groups for clients	Remote self-management counselling groups Support platforms Networks Disease and symptom targeted groups Caregiver targeted groups	
**Personal health tracking**			
	Access to own medical records	Mobile access to EMR^a^ or Kanta Services Access for caregivers	
	Self-monitoring of health or diagnostic data	Wearables and personal health tracking devices Connectivity to mobile client applications Feedback	
	Active data capture or documentation	Mobile or computer client application Checklists Diaries Symptom checkers Instruments	
**On-demand information services**			
	Look-up of health information	Mobile scanners Color coding of reference values Symptom checker and guidance Individual goals Activity tracking Tasks Feedback Rewarding	

^a^EMR: electronic medical record.

#### Targeted Client Communication

The subcategories of requirements for targeted client communication were inductively formed under 3 generic categories: health information, alerts and reminders, and diagnostic results. The DHIs should provide long-term access to multimodal (client applications, websites, or digital care pathways), evidence-based, CVD-related health, well-being, and service information before (primary prevention) and after CVD (follow-up). The health care professionals could assign clients (based on the personal health tracking data) to a suitable modality; prescribe tasks and activities; and provide counseling, education, and guidance to support the clients’ self-management, care, and rehabilitation. The DHIs should enable self-monitoring with visual feedback (eg, graphs and numeric data). The information should be provided conveniently and adaptively based on choices, as described by 2 participants.

You could use an app and one part of it could be counselling using video material. It would be good if that app could be used before getting sick and afterwards, so it could be versatile in that way.

Health care professional 4

…You could decide yourself when, at what moment you would use the app. Or when you have energy for rehabilitation...If you are tired when somebody is coming to counsel you, then you may not get everything out of it. You could time it better.

Patient 8

The transmission of health information should be tailored according to individual needs (eg, disease, personal challenges, risk factors, functioning capabilities, and stage of care pathway) and include audiovisual content (eg, pictures, comics, movies, and speech), as demonstrated by 1 participant.

If you want multimedia content, then some form of electronic application or a web page or something could be good in that sense. It could have videos and some spoken content on a subject… It would be good for those people whose comprehension has taken a hit… CVD can include visual field defects, or with older patients, there could be a cataract or something else…so something you can listen to would be really good.

Health care professional 13

Involving caregivers was important, with information to support and guide them on the CVD care pathway. Information should be provided on CVD-related changes in the patient’s mental health, personality, and functioning, as well as information on the availability of services (eg, peer-support networks). Caregivers should have information on how to support the patient, but also their own well-being.

Alerts and reminders should inform, recommend, and guide clients about their health status, risk factors, self-management (eg, medication, diet, exercise, and appointments), rehabilitation, and emergency situation (eg, acute CVD) assessment. This could be done by messaging color-coded reference values (eg, traffic lights) for their health data and visualization of the relation of their actions to risk reduction. The information could be personalized based on patient data in the form of games and personal tasks provided with customizable messaging intervals based on the client’s needs, as demonstrated by 2 participants.

It could have information such as CVD signs and reminders. It could be game-like, so for example it could test whether you identify things, and it could have tips on what to do if something happens.

Health care professional 5

Additionally, mobile access to long-term diagnostic results could help clients to follow their self-management. In addition, the DHIs should include alerts and reminders to inform caregivers of the client’s situation and of emergencies.

#### Untargeted Communication

The subcategories of requirements for untargeted communication were inductively formed under 1 generic category: untargeted health information. DHIs should provide general CVD-related information (eg, prevention, signs and symptoms) to the public via different multimodalities of 1-way communication (television, social media, and web-based materials).

#### Client-to-Client Communication

The subcategories of requirements for client-to-client communication were inductively formed under 1 generic category: peer groups for clients. Peer groups could be in the form of remote self-management (eg, smoking and weight) counseling groups, peer networks, and on platforms that enable the sharing of experiences. Peer groups could be targeted based on the client’s disease or symptoms to create group unison with their own peer support for caregivers.

#### Personal Health Tracking

The subcategories of requirements for personal health tracking were inductively formed under 3 generic categories: access to the patient’s own medical records, self-monitoring of health or diagnostic data, and active data capture and documentation. Access to the patient’s own health data at Kanta Services was important, but the DHIs should also provide mobile access to electronic medical records (EMRs) for clients and caregivers. The self-monitoring of health and diagnostic data (eg, heart rhythm, neurological status, sleep, physical activity and movement, blood pressure, and blood glucose) could be performed by wearables or personal health tracking devices with connectivity to their own client applications. Feedback from client applications could help the assessment and reaction to the patient’s own condition as described by 1 participant.

We should identify atrial fibrillations and other atrial arrhythmias…I think there could some kind of smart watch applications, that could have an alarm for those patients with an irregular heart rate.

Health care professional 5

Active data capture or documentation of personal health monitoring and self-management (eg, physical exercises, CVD risks, symptoms, pain, and sleep) data should be done using different client applications, checklists, diaries, symptom checkers, and instruments (ie, questionnaires) for clients to evaluate their situation, as described by 1 client.

…If there was some kind of easy to use application, for example a food diary, then you could follow it yourself, and check if you have stayed on it or not.

Patient 19

#### On-Demand Information Services

The subcategories of requirements for on-demand information services were inductively formed under 1 generic category of looking up health information. DHIs could provide support for clients’ decision-making and self-management. Mobile product scanners could be used to identify medications and foods with additional color coding (ie, traffic lights) indicating recommended food consumption. Features such as individual goal setting, personalized feedback, and rewards (points or material goods) for tasks and activities could support making better lifestyle choices (eg, sleep and exercise). These DHIs could also be useful to caregivers as family members might often have similar activity habits. Clients could use Q&A services with a health care professional or chatbot contact, and symptom checkers with visualization tools and alerts to support clients and caregivers in their help-seeking (eg, acute CVD and physical or mental health conditions), as demonstrated by 1 participant.

For the caregiver, there could be an application, for instance, showing whether there are signs of limb function, whether the mouth is drooping, or if there are signs of dizziness…It should be in plain language, with a design that immediately shows you the values as our NEWS score and what to do… For instance, it might tell you to immediately call an emergency number and so on.

Health care professional 4

### Health Care Professionals

The opportunities of DHIs for clients were classified under 6 main categories: client health records, decision support, telemedicine, training, prescription and medication management, and laboratory and diagnostics imaging management (Table 4).

**Table 4:** Identified opportunities and requirements of digital health interventions for health care professionals in CVD^a^ care.

Main category and generic category			Subcategory
**Client health records**			
	Longitudinal tracking of clients’ health status and services	Shared EMR^b^ for levels of care EMR integrated CVD registry Indicator data collection	
	Manage structured clinical records	Monitoring data transfer directly from devices to EMR	
	Manage unstructured clinical records	Capsulizing description	
	Routine health indicator data collection and management	Health measurement kiosks Electronic questionnaires Systematic data collection Targeted queries	
**Decision support**			
	Prompts and alerts	Reminders Notifications Guidelines Data visualization Data combination EMR integrations Mobile and computer access Scoring systems with alerts Real-time alerts	
	Checklist	Mobile and computer client applications EMR integration Part of patient data visualization Reminders	
	Screening	Triage Treatment selection Data combining Scoring tools Audio-visual recording and mediating Movement data with wearables Remote examination Mobile technology	
**Telemedicine**			
	Remote consultations	Videoconference Collaborative digital platforms Real-time chat Remote visits for caregivers Personal emergency response systems with client applications or remote alarm wristbands	
	Remote monitoring	Personal health monitoring device and wearable integrations Data transfer to EMR and/or cloud service Audio-visual recording Alerts to health care professional Automatic booking	
	Transmission of medical data	Low threshold client communication system Caregiver client communication system Referral requests Need assessment requests Appointment booking Messaging Health and activity data documentation Symptom diaries Digital care pathways and platforms	
	Case management between providers	Digital care pathways Networks Shared calendar Computer and mobile access Unofficial documentation platform Hybrid appointments Databases	
**Training**			
	Training content and reference material	Learning and training system Web-based learning Audio-visual material	
**Prescription and medication management**			
	Transmit or track prescription orders	Transmit purchase data from pharmacies to EMR	
**Laboratory and diagnostics imaging management**			
	Capture diagnostic results from digital devices	Mobile CT^c^ scan	

^a^CVD: cerebrovascular disease.

^b^EMR: electronic medical record.

^c^CT: computer tomography.

#### Client Health Records

The subcategories of requirements for client health records were inductively formed under 4 generic categories: longitudinal tracking of clients’ health status and services, managing structured clinical records, managing unstructured clinical records, and routine health indicator data collection and management. Longitudinal tracking of the client’s health status and services could be achieved by using a shared EMR on levels of care with an integrated CVD register and quality data collection as described by 1 participant.

An electronic medical record with an integrated CVD registry, where on follow up visits, you would enter data…

Health care professional 2

Structured clinical records would benefit from direct health monitoring data transfer from devices to EMRs, reducing manual and duplicate documentation. Unstructured clinical records could be managed with agreed upon capsulizing descriptions (eg, signs of CVD) to increase the comparability between documentation.

Routine health indicator data collection and management would benefit from patient-reported outcomes and self-management data (eg, lifestyle, well-being, and risk factors). The data collection method could include public health measurement kiosks and electronic questionnaires integrated with EMRs or delivered to smartphones. DHIs could ensure systematic data collection from targeted client groups, and this would also enable health care professionals to react to needs, as described by 1 participant.

A questionnaire that goes to certain patient group… for instance, for the next five years, or once a year. Also, if the users somehow report that things are not going well, then someone would receive that information and offer help. For example, if there has been something like six months or a year and the patient realizes that they are not getting better, then that would be a dangerous moment. That would be a good moment to offer help.

Health care professional 16

#### Decision Support

The subcategories of requirements for decision support were inductively formed under 3 generic categories: prompts and alerts, checklists, and screening by risk or other health status. Prompts and alerts could be provided via an EMR user interface on a computer or smartphone or to separate applications to health care professionals. Prompts and alerts could support planning, monitoring (eg, examination, CVD signs, and help seeking), and care and rehabilitation based on evidence-based guidelines and protocols and CVD service information, as described by 1 participant.

You would not have to rely on your own self-confidence. You would get support from the information technology…because sometimes it seems that people are scared to call in…There could be a protocol that says you need to call someone.

Health care professional 18

They could also be in the form of visual presentations or combinations of monitoring data and EMR data. Alert functions in real-time based on monitoring data, scoring systems, documentation, or combinations of these could be provided to support acute decision-making.

Checklists could be part of the user interface data visualizations in the EMR or separate mobile and computer client applications, and they could support patient counseling, evaluation (eg, for medical statements), care (eg, monitoring, examination, consultations, laboratory- and imaging needs, care tasks) and work shift–related duties, as described by 1 participant.

The insecurity, that have I done everything? What could be good is that, for example in the cardiac intensive unit, they have a feature in their own system, so that if you open a certain view, you get a list, where you tick everything off. You take into account everything that needs to be done for the patient’s recovery…

Health care professional 24

DHIs with tools for screening could combine data from various sources (eg, Kanta Services; monitoring, audiovisual, health, and indicator data; and data from scoring systems) for population or individual screening. They could be used for triage (eg, preventive care, follow-up, immediate diagnosis, treatment, and rehabilitation), to select between treatment or service options, and evaluate progression. Screening features could also include different mobile and wearable technologies to record and mediate audio-visual and movement data for remote examination, triaging and monitoring symptom progression (eg, aphasia or apraxia, neglect, and limb weakness) for decision-making in acute care, and rehabilitation in CVD care, as described by 1 participant.

Perhaps in places where a neurologist or a doctor with a neurology specialization are not available, then maybe some form of video imaging could be useful for assessing symptoms and deciding on the right treatment forms.

Health care professional 1

#### Telemedicine

The subcategories of requirements for telemedicine were inductively formed under 4 generic categories: remote consultations, remote monitoring, transmission of medical data, and case management between health care professionals. Remote consultations could be used to provide various CVD-related services synchronously to clients and caregivers such as emergency services, follow-up appointments, rehabilitation (eg, neurological testing and neuropsychological), counseling (eg, coaching and adaptation training), education (eg, CVD information and lectures) and social work services, as explained by 1 participant.

Perhaps there could be a videoconference option so that you could see the patient and the doctor or nurse could run the neurological tests that we do here. Perhaps then they could also be followed up via video, so there is no need to come here and wait.

Health care professional 18

In addition, caregivers from long distances could remotely visit clients and participate in ward rounds. Remote consultations could be in the form of videoconferencing, real-time chat services, collaborative digital platforms for filling and submitting administrative forms with assistance, or personal emergency response systems with mobile client applications or wristbands with remote alarms.

Remote monitoring DHIs could be used to transfer data (eg, on vital functions, blood glucose, and activity or movement) from personal or health care professionals prescribed health monitoring devices and wearables directly using integrations with cloud services or EMR. The wearables could also be sent for later analysis (ie, home telemetry) as a service. Remote monitoring could also be in the form of audio-visual monitoring combined with wearable data (eg, electrocardiogram and video monitoring of the facial area). With abnormal values, the DHI would automatically send an alert to the health care professional or emergency services or book an appointment for a follow-up, as described by 1 participant.

If somebody is paralyzed, they cannot move. There would be a device, for instance, that could send an alarm if the patient does not move for an hour. The patient would have given permission to use the device

Health care professional 5

The transmission of medical data included asynchronous messaging, referral and need assessment requests, appointment booking, and documentation of health data and activities (eg, self-monitoring values, symptom diaries) by the client from different health care professionals assigned client applications, platforms, and care pathways. The health care professional could contact relevant client groups (eg, based on risk factors and previous acute CVD) easily, to collect health and well-being data, and monitor the client’s health status and progress with the provision of information and guidance based on received data (see the *Clients* section). The possibility for a low threshold communication system for clients and caregivers to health care professionals was important, as described by 2 participants.

One idea could be a program and a connection to us. Then the patient could ask us questions, and the answer would come from here. There could be a specific time reserved to defuse and answer the questions.

Health care professional 9

There are many kinds of CVD-patients, and some might have communication difficulties and so on. In such cases, the caregiver could be involved as well. There could be a system for it, so that, if necessary, the patient or the caregiver could send a message, and someone could answer it…

Health care professional 14

The DHIs could enable better case management between health care professionals by adding mutual knowledge of available services and about the health care professional roles in the care and rehabilitation of CVD. Communication between health care professionals could be supported by computer and mobile access platforms (eg, shared digital calendars, networks, e-mails, and Microsoft Teams meetings). Health care professionals could jointly monitor and communicate on client progress, with possibilities for documenting unofficial information (eg, thoughts and conversations with clients) and hybrid appointments (eg, for functionality assessment), with other health care professionals participating remotely. There is a potential need for a national database for sharing knowledge on DHIs (ie, rehabilitation) and a potentially national CVD care pathway with the interprofessional collaboration of different health care professionals (organizations and levels of care), as described by 1 participant.

There is little information, that may not have updated, on the condition of the rehabilitation patient at the moment, so digital ways could be utilized with some care pathways. Professionals from different units could message better how the pathway has progressed.

Health care professional 21

#### Training

The subcategories of requirements for training were inductively formed under 1 main category: training content and reference material. The DHIs could provide continuous education on patient counseling via learning and training systems with web-based learning and audio-visual material to support the competence of health care professionals.

#### Prescription and Medication Management

The subcategories of requirements for prescription and medication management were inductively formed under 1 main category: transmitting or tracking prescription orders. DHIs could provide interoperability between pharmacy systems and EMRs in order to track prescribed medication purchases and provide indirect data on their consumption.

#### Laboratory and Diagnostics Imaging Management

The subcategories of requirements for laboratory and diagnostics imaging management were inductively formed under 1 main category: capturing diagnostic results from digital devices. DHIs could be in the form of mobile computer tomography scans.

### Data Services

The opportunities of DHIs for data services were classified under 3 main categories: data collection, management, and use; location mapping; and data exchange and interoperability (Table 5).

**Table 5:** Identified opportunities and requirements of digital health interventions for data services in cerebrovascular disease care.

Main category and generic category			Subcategory
**Data collection, management, and use**			
	Data synthesis and visualizations	Data mining from databases and EMRa Graphic presentations Compressed presentations Defined data presentation order Preference visualizations	
	Automated analysis of data	Speech analysis Integration to emergency services line Video analysis Audio-visual video modification Interpretation of CTb-scan images Prediction from data Data combination from databases and EMR	
**Location mapping**			
	Clients and households	Wearable sensors for location tracking	
**Data exchange and interoperability**			
	Data exchange across systems	Interoperability Applications Data exchange between governmental databases Interoperability between Kanta Services and local databases	

^a^EMR: electronic medical record.

^b^CT: computer tomography.

#### Data Collection, Management, and Use

The subcategories of requirements for data collection, management, and use were inductively formed under two categories: data synthesis and visualizations, and the automated analysis of data. Compressed and combined data visualizations could be created for the planning and assessment of care and rehabilitation with data mining from relevant databases and from the EMR sections to the user interface. The visualizations could provide data in a specific order (eg, chronological, clinical specialties), or based on health care professional preferences (eg, recanalization therapy indicators and contraindications, disease specificity, CVD signs, therapy evaluations) with quick retrieval. The DHI could also combine data (eg, medication and vital functions) for the assessment of treatment effects, as described by 1 participant.

The blood pressure and given medication could be presented side by side in the same view along with the administration route used. Then you could easily see what has led to a certain reaction, or what has lowered the blood pressure, what medication has been most effective, or if some medication has lowered the pulse too much, or whether it has been unsuitable for the patient.

Health care professional 1

The DHIs could provide automated analyses (eg, via machine learning or artificial intelligence [AI]) of different data sources to create new information to support decision-making. A DHI could analyze the patients’ speech to detect aphasia and dysarthria via integration with an emergency service line in acute CVD situations. The automated analysis of EKG (eg, for arrhythmias) and CT images could increase the quality of their interpretation. This could also combine and analyze data with predictive recommendations for care such as recovery prognoses, ideal rehabilitation timeframes, and risk assessments (postthrombolysis hemorrhage and deterioration). Predictive recommendations could also help in the treatment selection (eg, recanalization therapy, medication, and fluid therapy), as described by 1 participant.

A form of support could be something where you enter Stroke Scale scores and patient’s symptoms, and it would also ask contraindications and all that. The doctor would enter them there, and based on those, it would recommend thrombolysis or not.

Health care professional 2

Video analysis could be used to monitor patients’ progress in terms of CVD symptoms or rehabilitation effects. Modification of facial audio-visual video recordings could be used in client applications to visualize the effects of CVD signs for the client to increase awareness.

#### Location Mapping

The subcategories of requirements for location mapping were inductively formed under 1 main category: clients and households. DHIs could be in the form of wearable sensors that could be used to track the location of missing clients.

#### Data Exchange and Interoperability

The subcategories of requirements for data exchange and interoperability were inductively formed under 1 main category: data exchange across systems. The DHI should enable forms of interoperability between all health care providers on all levels, sectors, and districts of care, but also between governmental services (eg, tracking clients’ driving licenses and traffic violations). Better exchange of client and health data (eg, illnesses, recent treatments, medication, memory and cognition, motion and functioning abilities), rehabilitation feedback, and current caregivers’ contact information would be needed to keep them up to date and available at relevant stages of care. Interoperability was also found to be needed between Kanta Services and local databases of service providers.

## Discussion

### Principal Findings

This study identified several opportunities for DHIs from the primary users’ perspectives to address health system challenges in CVD, resulting in 14 main categories, 27 generic categories, and 126 subcategories, respectively. The identified opportunities for DHIs were related to information, availability, quality, acceptability, utilization, efficiency, and accountability of health systems.

The client and health care professional perspectives were studied together, with similar and complementary findings. The client and health care professional primary user categories both included opportunities and requirements for DHIs to support communication, data sharing (eg, monitoring with wearables, interoperability between systems, applications, and digital devices), and decision-making (eg, checklists and symptom checkers). Both categories also included requirements to support the delivery of information, such as data visualization, reminders, and alerts. These findings provide information for the development of DHIs with functionalities that support primary user-centered and beneficial health systems.

### Comparison With Prior Work

For clients, the identified opportunities for DHIs were related to information (eg, lack of quality or reliable data, lack of access to information or data, and insufficient utilization of data and information), quality (eg, inadequate supportive supervision), acceptability (eg, programs that do not address individual beliefs and practices), and accountability (eg, insufficient patient engagement). In line with the prior literature, there is a lack of quality or reliable data from both clients’ and caregivers’ perspectives [11,12], while client applications and digital care pathways could improve the access to information or data [18] to support self-care (eg, tracking and decision-making) from primary to secondary prevention and beyond. However, attention should be paid to the provision (eg, tailoring, timing, accessibility, and multimedia) of information to address individual health needs and practices [15,17]. The culture, language, and literacy of clients should also be considered when deploying DHIs in CVD care to increase acceptability [25].

Clients with CVD need access to EMRs to manage their own health data [18]. This study elaborates that clients need to be able to access and use information (eg, laboratory values) as well as behavioral, health, and well-being data, along with decision-support tools (eg, symptom checkers and checklists). Furthermore, there is a need for self-monitoring and documentation supporting engagement for self-management before and after an acute CVD to reduce the occurrence [38], recurrence [39], health care costs [7] of CVD, and resource use [40] in health care.

In line with the previous literature, there is a lack of supportive supervision for caregivers [11,12]. Correspondingly, client applications and communication systems are rarely used by caregivers [41]. According to this study, DHIs could offer information and communication systems to caregivers, highlighting the need to support caregivers in digitalization. Interestingly, the need for mutual client and caregiver DHIs was also expressed, which could be beneficial as psychoeducational interventions engaging both client groups have been found to relieve the caregiver burden, and improve the patient’s quality of life, functional independence, family functioning, and health care service use [42], revealing a valid area for future research and development.

For health care professionals, the identified opportunities for DHIs were related to information (eg, lack of quality or reliable data, communication roadblocks), quality (eg, insufficient continuity of care, health worker competence, poor adherence to guidelines), efficiency (eg, inadequate workflow management, poor planning, coordination of care), and accountability (eg, poor accountability between the levels of the health sector).

There is a lack of quality data, where registries and electronic questionnaires could be used to collect data (eg, patient-reported outcome and self-management data), to support benchmarking of CVD care for the monitoring and improvement of stroke services nationally [43] and increase accountability. In addition, health kiosks [44] could be implemented for non–tech-savvy individuals (lack of device or digital literacy) to collect clinical measurements for population information. Remote monitoring (eg, wearables and integrations) of traditional and novel measurement values (eg, oxygen saturation, electroencephalogram, and movement) with functions to alert health care professionals, could improve access to reliable health data, increase the quality of follow-up and thus, reduce health care use, hospitalization, and mortality [45,46].

In line with previous research [11,21], there were communication roadblocks between clients, caregivers, and health care professionals due to traveling distances and lack of contact information, along with the lack of planning and coordination of care after acute CVD [8,9]. Telemedicine (eg, telerehabilitation, follow-up, and digital counseling) could provide low-threshold client communication systems as well as intersectoral and professional collaboration platforms to increase coordination and continuity of care. According to the participants, digital care pathways could provide combined systems for monitoring, communication, and bringing together the relevant health care professionals for service provision in CVD care.

The need to support competence and adherence to guidelines along with improvements in workflow management were identified. In line with the previous literature [47-49], the participants in this study identified the need for digital decision-support tools, especially in the prehospital and acute phase of CVD to screen (triage), perform acute diagnoses, select treatment, and monitor symptoms and conditions. Supportive features such as information provision, instructional guidance, and client-specific data were included in the decision-support tools which are associated with successful technological interventions [50]. Despite their acceptability, there exists a lack of evidence on the effectiveness of support systems for clinical decision-making, warranting further research [51].

For data services, the identified opportunities were related to information (eg, insufficient utilization use of information or data) and efficiency (eg, poor planning and coordination, and delayed provision of care). Poor planning and coordination were identified in CVD care, with requirements of DHIs highlighting the importance of interoperable EMRs and health management information systems on all levels and sectors of health care, as functioning health information exchange can secure the coordination of care and improve the quality of care and health outcomes [52,53]. In addition, traditional and novel (eg, video and speech analysis) data sources could be used with AI, machine learning, and data mining technologies to create predictive data on the course and outcomes of CVD [54,55]. This could accelerate decision-making and planning for the efficient provision of care. As there is limited data on the clinical applicability of AI solutions in CVD care, the primary user requirements of this study could be used along with rigorous validation research to support need-based development, as AI has the potential to improve health outcomes [54].

### Strengths and Limitations

This study has some limitations. The results may not be transferable to all CVD care contexts, as the clients in this study were patients with ischemic stroke and TIA, and health care professionals worked mainly in the acute hospital care of these patients. The clients were also interviewed in acute phases of their CVD care which could affect their cognition and perceptions. DHIs were not identified for all of the WHO categories, and especially not for the health system managers’ primary user group. This could be due to the study not targeting health care managers and that the interview topic guides were not designed according to the WHO categorization. On the other hand, the results of this study were similar to previous studies, adding credibility. The foremost strength of this study is the combination of the client and health care professional perspectives to identify DHIs suited for primary user needs.

### Conclusions

This study identified several opportunities for DHIs to address health system challenges in CVD care. The identified opportunities were related to information, availability, quality, acceptability, utilization, efficiency, and accountability. DHIs are needed to provide long-term reliable health information, support, and communication possibilities to clients and their caregivers. Health care professionals can benefit from DHIs that provide access to relevant data to inform care and clinical decision-making with the support of interdisciplinary work in the care of CVD on all levels and sectors of health care. These opportunities and requirements provide valuable insights into the digital transformation and thus, the emerging use of digital technologies for health in cerebrovascular care.

## Appendix

Multimedia Appendix 1 Consolidated Criteria for Reporting Qualitative Research (COREQ) checklist.

## Abbreviations

AI: artificial intelligence
COREQ: Consolidated Criteria for Reporting Qualitative Research
CVD: cerebrovascular disease
DHI: digital health intervention
EMR: electronic medical record
TIA: transient ischemic attack
WHO: World Health Organization

## Data Availability

The datasets generated and/or analyzed during this study are not publicly available due to research permit restrictions, but the corresponding author can be contacted for more information about the study.
