# Supplementary material for: Perspectives of Clients and Health Care Professionals on the Opportunities for Digital Health Interventions in Cerebrovascular Disease Care: Qualitative Descriptive Study
Source: J Med Internet Res. 2024 Dec 2;26:e52715. doi: 10.2196/52715 (PMC11650084; doi:10.2196/52715)
Supplement: Multimedia Appendix 1 [file jmir_v26i1e52715_app1.docx]

**Consolidated Criteria for Reporting Qualitative Research (COREQ): 32-item checklist.**

Based on: Tong A, Sainsbury P, Craig J. Consolidated criteria for reporting qualitative research (COREQ): a 32-item checklist for interviews and focus groups. International Journal for Quality in Health Care. 2007. Volume 19, Number 6: pp. 349 – 357.

| **Topic** | **Item No.** | **Guide Questions/Description** | **Reported on Page No.** |
| --- | --- | --- | --- |
| **Domain 1: Research team and reflexivity** | | | |
| ***Personal characteristics*** | | | |
| Interviewer/facilitator | 1 | Which author/s conducted the interview or focus group? | 3 |
| Credentials | 2 | What were the researcher’s credentials? E.g., PhD, MD | 4 |
| Occupation | 3 | What was their occupation at the time of the study? | 4 |
| Gender | 4 | Was the researcher male or female? | - |
| Experience and training | 5 | What experience or training did the researcher have? | 4 |
| ***Relationship with participants*** | | | |
| Relationship established | 6 | Was a relationship established prior to study commencement? | 5 |
| Participant knowledge of the interviewer | 7 | What did the participants know about the researcher? e.g., personal goals, reasons for doing the research | - |
| Interviewer characteristics | 8 | What characteristics were reported about the inter viewer/facilitator? e.g., Bias, assumptions, reasons and interests in the research topic | - |
| **Domain 2: Study design** | | | |
| ***Theoretical framework*** | | | |
| Methodological orientation and Theory | 9 | What methodological orientation was stated to underpin the study? e.g., grounded theory, discourse analysis, ethnography, phenomenology, content analysis | 3 |
| ***Participant selection*** | | | |
| Sampling | 10 | How were participants selected? e.g., purposive, convenience, consecutive, snowball | 3 |
| Method of approach | 11 | How were participants approached? e.g., face-to-face, telephone, mail, email | - |
| Sample size | 12 | How many participants were in the study? | 5-6 |
| Non-participation | 13 | How many people refused to participate or dropped out? Reasons? | 3 |
| ***Setting*** | | | |
| Setting of data collection | 14 | Where was the data collected? e.g., home, clinic, workplace | 3 |
| Presence of non-participants | 15 | Was anyone else present besides the participants and researchers? | 3 |
| Description of sample | 16 | What are the important characteristics of the sample? e.g., demographic data, date | 5-6 |
| ***Data collection*** | | | |
| Interview guide | 17 | Were questions, prompts, guides provided by the authors? Was it pilot tested? | 3-4 |
| Repeat interviews | 18 | Were repeat inter views carried out? If yes, how many? | 3-4 |
| Audio/visual recording | 19 | Did the research use audio or visual recording to collect the data? | 3-4 |
| Field notes | 20 | Were field notes made during and/or after the interview or focus group? | 3-4 |
| Duration | 21 | What was the duration of the interviews or focus group? | 3-4 |
| Data saturation | 22 | Was data saturation discussed? | 3-4 |
| Transcripts returned | 23 | Were transcripts returned to participants for comment and/or | - |
| **Domain 3: analysis and findings** | | | |
| ***Data analysis*** | | | |
| Number of data coders | 24 | How many data coders coded the data? | 4 |
| Description of the coding tree | 25 | Did authors provide a description of the coding tree? | 4 |
| Derivation of themes | 26 | Were themes identified in advance or derived from the data? | 4 |
| Software | 27 | What software, if applicable, was used to manage the data? | 4 |
| Participant checking | 28 | Did participants provide feedback on the findings? | - |
| ***Reporting*** | | | |
| Quotations presented | 29 | Were participant quotations presented to illustrate the themes/findings?  Was each quotation identified? e.g., participant number | 6-16 |
| Data and findings consistent | 30 | Was there consistency between the data presented and the findings? | 6-18 |
| Clarity of major themes | 31 | Were major themes clearly presented in the findings? | 6-18 |
| Clarity of minor themes | 32 | Is there a description of diverse cases or discussion of minor themes? | - |
